# The ethics of community-based research with people who use drugs: results of a scoping review

**DOI:** 10.1186/s12910-016-0108-2

**Published:** 2016-04-29

**Authors:** Rusty Souleymanov, Dario Kuzmanović, Zack Marshall, Ayden I. Scheim, Mikiki Mikiki, Catherine Worthington, Margaret (Peggy) Millson

**Affiliations:** Factor-Inwentash Faculty of Social Work, University of Toronto, 246 Bloor St. W., Toronto, ON M5S 1V4 Canada; Joint Centre for Bioethics, University of Toronto, 155 College Street, Toronto, ON M5T 1P8 Canada; Division of Community Health & Humanities, Health Sciences Centre, Faculty of Medicine, Memorial University, 300 Prince Philip Drive, St. John’s, NL A1B 3V6 Canada; Department of Epidemiology and Biostatistics, Schulich School of Medicine and Dentistry, The University of Western Ontario, K201 Kresge Building, London, ON N6A 5C1 Canada; Harm Reduction Peer Street Outreach Coordinator, Queen West Central Toronto Community Health Centre, 168 Bathurst Street, Toronto, ON M5V 2R4 Canada; School of Public Health and Social Policy, Faculty of Human and Social Development, University of Victoria, HSD building, Victoria, BC V8W 2Y2 Canada; Dalla Lana School of Public Health, University of Toronto, 155 College Street, Toronto, ON M5T 1P8 Canada

**Keywords:** Scoping review, Community-based participatory research, Ethics, People who use drugs, Challenges, Incentives, Exclusion, Diversity

## Abstract

**Background:**

Drug user networks and community-based organizations advocate for greater, meaningful involvement of people with lived experience of drug use in research, programs and services, and policy initiatives. Community-based approaches to research provide an opportunity to engage people who use drugs in all stages of the research process. Conducting community-based participatory research (CBPR) with people who use drugs has its own ethical challenges that are not necessarily acknowledged or supported by institutional ethics review boards. We conducted a scoping review to identify ethical issues in CBPR with people who use drugs that were documented in peer-reviewed and grey literature.

**Methods:**

The search strategy focused on three areas; community-based research, ethical issues, and drug use. Searches of five academic databases were conducted in addition to a grey literature search, hand-searching, and consultation with organizational partners and key stakeholders. Peer reviewed literature and community reports published in English between 1985 and 2013 were included, with initial screening conducted by two reviewers.

**Results:**

The search strategy produced a total of 874 references. Twenty-five references met the inclusion criteria and were included in our thematic analysis. Five areas were identified as important to the ethics of CBPR with people who use drugs: 1) participant compensation, 2) drug user perspectives on CBPR, 3) peer recruitment and representation in CBPR, 4) capacity building, and 5) participation and inclusion in CBPR.

**Conclusions:**

We critically discuss implications of the emerging research in this field and provide suggestions for future research and practice.

## Background

Drug user networks and community-based organizations have underlined the importance of greater, meaningful involvement of people who use drugs in research, community programs and services, and policy initiatives [1, 2]. Embracing the values of “Nothing About Us, Without Us” [3], we are called on to acknowledge histories of research exploitation [4] and to better engage and support the leadership and involvement of people who use drugs [5–7].

Conducting community-based participatory research^1^ (CBPR) in partnership with people who use drugs is one approach to supporting this engagement strategy, along with capacity-building among community and academic partners. CBPR can produce community-relevant results that are directly applicable to programs, policy, and practice [8]. CBPR is a collaborative approach to conducting research where multiple stakeholders are equitably involved in all stages of the research process [9]. In North America, Oceania, Europe, and East and South East Asia, we have seen an increase in community-based research projects that include drug users across a range of roles from study design, to recruitment, data collection and analysis, and knowledge dissemination [10].

Alongside the increased use of CBPR are dialogues concerning the ethical challenges with this form of research [11, 12]. While CBPR shares some aspects of more basic scientific inquiry, there are marked differences including approaches that value close connections with community organizations and participants [13], the engagement of people with lived experience in recruitment and data collection [14, 15], and data analysis strategies that involve community members with access (or modified access) to what may be sensitive and confidential information [16]. Each aspect of these partnerships poses benefits and potential drawbacks and underlines the importance of attending to complicated power dynamics, unequal access to resources, and differing stakes in the process and outcomes of the research endeavour [17–19].

Research ethics boards or institutional review boards^2^ (REBs or IRBs) that were first developed to respond to the ethical challenges of research in the basic or clinical sciences continue to expand their understanding of CBPR and the best strategies to support more ethical forms of participatory research [20]. Where REBs may be tasked with gatekeeping roles in more traditional research, their lack of familiarity with community-based research may lead them to overemphasize those aspects of the ethics approval process that are more appropriate to basic science or clinical trials [21, 22] while overlooking or failing to attend to potential research challenges in complex community settings and partnerships [20].

The research team for this project is made up of people who have experience conducting CBPR with people who use drugs, researchers with experience and expertise in research ethics, and people who use drugs. Our goals were to identify ethical issues in community-based research with people who use drugs that were documented in the literature and to create a knowledge dissemination tool to share this information with drug users and community organizations working with people who use drugs (for more information on this resource, see http://drugscbrethics.com). In this paper, we report on the results of the scoping review, highlighting key ethical challenges, promising practices, and areas where further research is recommended. No personal or sensitive data was collected for this project. University of Toronto Research Ethics Board deemed this projected not to require ethics review^3^

## Methods

### Scoping review methodology

The methodology for this scoping review followed systematic steps to identify, gather, summarize, and integrate large numbers of individual studies on the topic of CBPR ethics with people who use drugs. We used Levac, Colquhoun, and O’Brien’s [23] definition of a scoping review as, “a process of summarizing a range of evidence in order to convey the breadth and depth of a field” [23]. Scoping reviews allow researchers to rapidly map the key concepts and the main sources of evidence underpinning the research area [24].

The main steps of this scoping review included an initial search for relevant peer-reviewed and grey literature, screening for relevant studies that met pre-determined inclusion and exclusion criteria established by the research team, data extraction, and thematic analysis. Although presented as a series of steps, the process was iterative, where some steps were repeated when necessary to ensure that a broad range of literature was included. Below we summarize the methodology for this paper in detail using the Arksey and O’Malley Framework [24].

## Framework stage 1: identifying the research question

The objectives of this scoping review were: 1) to summarize research findings on CBPR ethics and practices with people who use drugs; and, 2) to identify gaps in the existing research literature and opportunities for future research focused on ethical issues. In order to meet these objectives, we conducted a scoping review of empirical studies and grey literature published between 1985 and 2013 to identify published work across various disciplines pertaining to CBPR ethics with people who use drugs. We selected 1985 as a starting date due to the increase in publications related to CBPR after this time. Our scoping review was guided by the question: “What ethical issues in CBPR with people who use drugs are documented in peer-reviewed and grey literature?”

## Framework stage 2: identifying relevant studies

### Search strategy

In order to identify relevant studies, a computerized search was conducted of the following databases: 1) ProQuest (Applied Social Sciences Index and Abstracts, Biological Sciences: Virology and AIDS Abstracts, Canadian Research Index, ERIC, ProQuest Research Library, ProQuest Science Journals, PsycARTICLES, PsycCRITIQUES, PsycINFO, Social Services Abstracts, Sociological Abstracts); 2) Ovid **(**Ovid MEDLINE, Embase, NASW Clinical Register, Social Work Abstracts); 3) JSTOR; 4) EBSCO host; and 5) Cochrane Library. For the current study, all databases were investigated over a 28-year period (1985–2013).

A librarian from the University of Toronto consulted on the development of our search strategy. The goal was to identify peer-reviewed and grey literature related to the ethics of community-based research with people who use drugs. For convenience, the search terms were divided into three foci: 1) for CBPR we selected terms inclusive to all types of CBPR (e.g., participatory, collaborative, action research, community-driven research) in order to accurately reflect variations in CBPR terminology; 2) in the “drug/substance use” category, terms were chosen to reflect the diverse nature of people who use drugs and the substances that may be used (e.g., injection drug use, amphetamines, crack, addiction), and; 3) the terms selected for the domain of “ethics” included words such as “guidelines”, “principles”, “best practices”, and “decision-making”. The search terms used are summarized in Table 1.

**Table 1:** Search terms

Community based research	Drug/substance use	Ethics
CBR OR CBPR OR Community-Based Research* OR Community-based adj2 research* OR Cooperative behav* OR Community adj2 research* OR Participatory adj2 research* OR Collaborative research* OR Community-engaged research* OR Action research* OR Action Science OR Community Development* OR Community Organi* OR Community Partner* OR Community Particip* OR Community-led research OR Community-led OR User-led adj2 research OR User-led OR Community Collaborat* OR Collaborative inquiry OR Community Involve* OR Involving Communit* OR Community Empower* OR Empowering Communit* OR Community driven OR Community-driven OR consumer involve* OR involving consumer OR community action OR Community-action	Drug use* OR Using drugs OR drug-using OR drug using OR Substance use* OR substance-using OR substance using OR Drug abuse* OR drug-abusing OR Drug misuse* OR user* OR Substance abus* OR Substance-abusing OR Drug dependence OR Substance dependen* OR Addict* OR Drug addict* OR Injection adj2 use* OR Injection use* OR Drug injection* OR People adj2 inject OR People adj2 drugs OR Intravenous adj2 use* OR Intravenous adj2 abuse* OR illicit adj2 use* OR Illicit drug* OR Illicit use* OR PWUID OR PWUID OR people adj2 crack OR Heroin use* OR Heroin-use* OR Heroin OR Crack use* OR Crack-use* OR crack OR Cocaine us* OR Cocaine-using OR cocaine OR Meth use* OR meth OR Amphetamine us* OR Amphetamine-us* OR Amphetamine OR Street drug* OR Needle shar* OR Needle-shar* OR Needle-exchange OR Needle exchange OR Parenteral adj2 use* OR Drug overdos* OR overdos* OR Substance-related OR Substance related OR Drug-related OR Drug related	Ethic* OR Ethics code OR Ethical Guidelines OR ethical standards OR ethical principles OR moral ethics OR Institutional ethics OR research ethics OR Bioethic* OR Best Practice* OR Conduct OR Code adj2 ethics OR Ethical issue* OR Responsibilit* OR Human Subjects OR Human Rights OR right* Ethic* adj2 research* OR Principle-based ethics OR prejudice OR stigma* disadvantage* OR marginal* OR discriminat* OR trust OR autonom* OR decision-mak* OR consent* OR confidential* OR legal liability OR financial compensation* OR incentive* OR reimbursement* OR recruitment

### Hand-searching

When a specific journal was identified as being key to finding relevant articles, it was hand-searched to identify articles in the relevant subject areas. In addition, authors and reference lists from papers identified as relevant were used to locate further references of interest. Further, we conducted an Internet search on Google using the same search strategy that was used in the academic databases. Grey literature included research, government or community reports, but excluded book chapters and policy documents.

### Existing organizations and networks

Additionally, we conducted a literature search (e.g., community reports) on the following organizational websites: Canadian Harm Reduction Network (http://canadianharmreduction.com), Vancouver Area Network of Drug Users (http://www.vandu.org), CACTUS Montréal (http://www.cactusmontreal.org), and Drug User Advocacy League (http://dualottawa.ca).

## Framework stage 3: study selection

### Inclusion criteria

For the purposes of this scoping review, full-length, empirical research articles and reports focusing on CBPR ethics with people who use drugs were included. The following inclusion criteria were identified: 1) published between 1985 and 2013; 2) English language articles from national and international peer-reviewed or scholarly journals, or reports dedicated to health, medicine, social sciences, and interdisciplinary research; 3) articles or reports about drug use/users; 4) articles or reports on the topic of “ethics” (including such diverse aspects of ethics as challenges, compensation, recruitment, wise practices, and empowerment), and 5) articles/reports on CBPR. References needed to meet all of the inclusion criteria in order to be included in the review. Book chapters and policy documents were excluded from the review.

Further, we excluded biomedical research with people who use drugs.

**Fig. 1:**
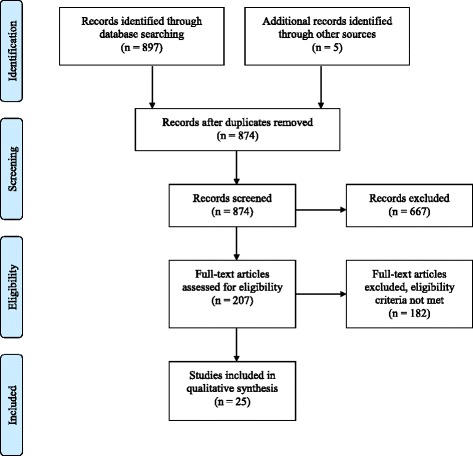
Flowchart of screening process

Titles and abstracts of all publications were imported into the data management software, Refworks, and subsequently exported into Excel for screening. First, one of the authors (RS) conducted the initial appraisal (Level 1 screen), where we identified potentially relevant publications. If the relevance of the study was not apparent after review of the article’s title and abstract, the full text was retrieved for screening in Level 2. During Level 2 screening, full articles and reports were closely examined and appraised to determine whether they met all pre-determined inclusion and exclusion criteria (Fig. 1). All seven authors participated in Level 2 screening, with two reviewers per article. Disagreements were resolved by a third reviewer.

## Framework stage 4: charting the data

After the authors conducted a detailed review of the full text of the article using the inclusion/exclusion criteria (see above), a data charting process was developed collaboratively by the team. Twenty-five references met the inclusion criteria and proceeded to the data charting stage. All seven authors participated in the data collection process. Each author cross-checked charted data with the other six authors for accuracy. In order to chart the data, the authors extracted the following information from the literature: 1) source (author, year of publication, title, and journal); 2) description of a population and setting (demographics of study participants, location of research, type of setting); 3) CBPR-specific characteristics, such as research procedure, design, recruitment of participants, data collection and data analysis, and; 4) Study findings and implications.

## Framework stage 5: collating, summarizing, and reporting the results

The process of collating and summarizing studies was guided by focusing on the examination of best practices and challenges when conducting CBPR with people who use drugs.

The main task involved drawing inferences from the literature. This followed an inductive analysis and was closely grounded in our research question. We compared all data and grouped similar concepts (e.g., compensation, capacity building, ethical challenges) from the literature into categories (sometimes one study fell in more than one category) and differentiated those concepts from one another. Based on this approach, we identified themes, which describe the findings we summarized under each category. Peer debriefing was also employed, in which all of the authors met regularly to discuss the data analysis process and to resolve any discrepancies. Five themes presented below were identified as important to the ethics of CBPR with people who use drugs.

## Results

A total of 897 references were retrieved from the database search, and 5 references from hand searching and organizational networks. During the initial appraisal we excluded 28 duplicates. Following this, we excluded 667 publications and identified 207 publications as potentially relevant (Level 1 screen; *Kappa* = 0.85). We excluded 667 publications because they did not meet all three inclusion criteria (e.g., the publication either did not focus on people who use drugs, and/or did not describe CBPR and/or ethical aspects of research).

Following this, the 207 publications that met the inclusion criteria based on title and abstract were retrieved for screening on full text (Level 2 screen; *Kappa* = 0.94). During this stage, full articles and reports were closely examined and carefully appraised for their relevance. After Level 2 screening, 182 publications were excluded from our review. Upon closer examination, 78 publications still did not meet at least two of the three inclusion criteria and were excluded.

Sixty-eight out of the excluded 182 publications did not specifically pertain to any ethical aspects of research, but rather explored topics other than ethical conduct in research. Twenty-nine of the excluded 182 publications did not pertain to community-based research or practice, but rather focused on other forms of research (e.g., biomedical or experimental research). The remaining eleven out of the 182 publications were excluded because the publications did not focus on people who use drugs. Only two publications [25, 26] that did not meet one of the three inclusion criteria (focus on CBPR) were still included in our review because they were deemed important by the reviewers and discussed essential aspects of research ethics with people who use drugs that could be easily applied to CBPR.

After the second screen, we were left with a total of 25 publications including 20 from academic databases, three from hand searching, and two publications from organizational networks, published between 1985 and 2013, the content of which is presented in Table 2. The studies reported in the literature were conducted in the following countries: Canada, the United States, the United Kingdom, and Australia. Based on the thematic analysis, we grouped the results into five areas: 1) participant compensation and retention, 2) perspectives of people who use drugs on CBPR, 3) peer recruitment and representation, 4) capacity building, and 5) participation and inclusion in CBPR.

**Table 2:** Retrieved studies (n = 25)

Theme	Citation
Participant compensation and retention in research	COCQ-SIDA (2012)
	Draus et al. (2005) ^a^
	Festinger et al. (2008)
	Salmon et al. (2010) ^a^
	Seddon (2005)
	Striley (2011) ^a^
	Striley et al. (2008) ^a^
	Wolfe and Cohen (2010) ^a^
Drug user perspectives on CBPR	Barratt et al. (2007)
	Draus et al. (2005) ^a^
	Fisher et al. (2008)
	Fry and Dwyer (2001)
	NAOMI Patients Association and Boyd (2012)
	Singer et al. (2008) ^a^
	Wolfe and Cohen (2010) ^a^
Peer recruitment and representation in CBPR	Baldwin et al. (2009) ^a^
	Brown et al. (2005) ^a^
	Cunningham-Williams et al. (1999)
	Perez et al. (2009)
	Rudolph et al. (2010) ^a^
	Salmon et al. (2010) ^a^
	Shannon et al. (2007) ^a^
	Singer et al. (2008) ^a^
	Sterk (1999) ^a^
	Stewart et al. (2012)
	Striley (2011) ^a^
Capacity building	Baldwin et al. (2009) ^a^
	Brown et al. (2005) ^a^
	Salmon et al. (2010) ^a^
	Shannon et al. (2007) ^a^
	Weeks et al. (2006)
Pragmatics of participation and social inclusion in CBPR	Aldridge and Charles (2008)
	DuBois et al. (2011)
	Levine et al. (1991)
	Rudolph et al. (2010) ^a^
	Salmon et al. (2010) ^a^
	Sterk (1999) ^a^
	Striley et al. (2008) ^a^

^a^Note: study classified under more than one theme

## Theme 1: participant compensation and retention in research

Participant compensation garnered most attention in studies we reviewed. Eight of the 25 included studies explored the ethics of compensation and incentive payments. Table 3 presents this literature by the types of questions raised with regard to fair compensation practices in research endeavors with people who use drugs.

**Table 3:** Research focus in studies discussing participant compensation and retention

Citation	Focus
COCQ-SIDA [31]	The importance of effective communication with regards to compensation in research.
Draus et al. [27]	The benefits of using cash payments (as opposed to other forms of compensation) to remunerate participants in research.
Festinger et al. [33]	The benefits of incentive payments to increase the quality of survey answers and facilitate access to people who are normally harder to reach.
Salmon et al. [32]	The importance of clarifying rules for what type of work receives honorarium (and its amount).
Seddon [28]	The decision-making process by researchers who do not offer compensation based on the assumption that participants use research incentives to purchase illegal drugs.
Striley [29]	The benefits of cash payments to increase the retention of people who use drugs in research.
Striley et al. [30]	The decision-making process by researchers and research ethics boards who do not compensate people who use drugs for their time with cash.

Within the seven studies on participant compensation and retention, four studies [27–30] place emphasis on the importance of fair compensation with cash payments as opposed to other forms of compensation (e.g., gift cards). This emerged as the most common topic of discussion in studies that we reviewed. Within this work, researchers discussed that not offering incentives and denying rewards (or offering incentive payments in the form of cash or vouchers) raises the question of whether drug users are treated the same way as other participants (or not) simply because of who they are [28]. One study discussed how often research ethics committees and boards may require that investigators not provide remuneration to drug-using participants, believing that those with a history of drug use may use study remuneration to buy drugs [30]. The consensus across studies is that people who (are known to) use drugs should not be compensated differently than non-users (e.g., cash when appropriate).

Two publications [31, 32] discuss the importance of effective communication with regards to various practical issues (e.g., rules) surrounding the compensation of research participants who use drugs. Here, publications discuss how effective communication by research organizations about the use of incentives to all relevant stakeholders in the community is essential [31], and should take place at the start of the research [28].

Three studies [28, 29, 33] explored how compensating participants with cash (as opposed to other forms of compensation) improves both the quality of research and the retention of people who use drugs in research. Research showed that neither the amount of compensation nor the mode of payment (cash or gift card) had a significant effect on new drug use among clients in an outpatient substance abuse treatment program; instead, individuals receiving cash (vs. gift cards) were more likely to use their payments for essential, non-luxury purchases [33]. Further, one publication [27] provided a discussion of the benefits of using cash payments over other types of remuneration, including the suggestion that the use of cash as payment for participation affirms the non-judgmental stance of the research project, maximizes the appeal of research participation, and minimizes the traceability of incentives (thereby protecting confidentiality).

Within this theme, four publications provided explicit recommendations. Striley [30] proposed asking participants what benefit they seek from participating in a community-based study (as any understanding of remuneration or benefit must be population-specific), and what remuneration they perceive as appropriate. Salmon and colleagues [32] suggest that the rules for what type of work receives honorarium and its amount should be fair and ensure that opportunities to receive compensation are clearly identified and equally divided among participants. Similarly, two publications [28, 31] recommend that effective communication by research organizations about the use of incentives to all relevant stakeholders in the community is essential and should take place at the beginning of the research.

## Theme 2: how people who use drugs view research practices

Six out of 25 publications highlight the nuances of how people who use drugs view research practices. These studies included discussions regarding participants’ perspectives and motivations to participate in research, and how past experiences influence participant views on research initiatives with people who use drugs. The overall findings in two publications [34, 35] were that motivations for participation in research are multi-dimensional, including a desire to provide and share information or expertise; participate in informed decision-making; express opinions; contribute to solving drug problems; help without consideration of personal gain; dispel myths about drug users; and improve services for drug users [35]. Similarly, another study [34] showed that when injecting drug users were asked to nominate the best and worst aspects of participating in research, benefits to others (assisting fellow drug users, provision of information, policy contributions) and personal benefits (cash payments, opportunities to talk and share experience) were identified as positive factors.

Table 4 reports various environmental and institutional factors, which were identified as negative aspects of participating in research by people who use drugs or as barriers to involvement of people who use drugs in research. Six publications explored the concerns of people who use drugs when participating in research. Three publications focused specifically on CBPR practices [27, 34, 36], while one publication focused on clinical trials with people who use drugs [25]. One publication [34] described the negative factors that hinder drug user involvement in research and identified how personal discomfort with research, inconvenience, and risk work as participation barriers for people who use drugs. Two studies [27, 37] highlighted that in places (e.g., smaller towns), where treatment programs and local criminal courts may be directly affiliated, research participants raise concerns that the information about ongoing drug use could be shared with treatment agencies and somehow make its way to law enforcement. Similar concerns with regard to experimental mistrust, fears of exploitation, and objectification at the hands of investigators are voiced by people who use drugs when participating in randomized control trials [25]. This theme was also salient in a community report [4], which describes how participants often link research with their personal experience of unfavorable and exploitative medical care, and distrust of clinical researchers. One study [34] identified perceived lack of impact of research findings as one of the worst things about research projects, and highlighted the importance of better communication by investigators as to how a study aims to impact policies and practices to the benefit of drug-user communities.

**Table 4:** Negative aspects of research participation, as identified by people who use drugs

Citation	Negative aspects of research
Barratt et al. [34]	Discomfort with research; inconvenience; risk; perceived lack of impact of research findings.
Barratt et al. [34]	Confidentiality and safety concerns, in particular due to criminalization of drug use.
Draus et al. [27]	
Wolfe and Cohen [37]	
Singer et al. [36]	
Barratt et al. [34]	Mistrust of experiments; fears of exploitation and objectification at the hands of investigators.
Fisher et al. [25]	
NAOMI and Boyd [4]	
Singer et al. [36]	Stigma related to HIV/AIDS or drug use; fear of family or community rejection; misconceptions and stereotypes about drug users.

## Theme 3: peer recruitment and representation in CBPR

Overall, peer recruitment is presented as a useful approach in six out of the 25 publications we reviewed [29, 38–42]. Various forms of peer recruitment are commonly recommended to improve enrolment of diverse samples of drug users to broaden the composition of participants in CBPR, including social network tracking and the utilization of peer referrals [38]; employing peers within a community as outreach workers [39]; recruitment through community partners (e.g., staff at local pharmacies) [40]; the use of community-peer hiring panels [41]; and the use of “translators” (i.e., individuals familiar with the cultures and language of street-involved active drug users, as well as academic researchers) [42]. Two publications [29, 39] clearly articulated that peer recruitment can reach community members using an insider approach, accessing more hard-to-reach groups, and increasing the generalizability of CBPR.

However, studies also cautioned researchers about the problematic consequences of using peer recruitment. For instance, one study [38] suggested that peer workers tend to successfully recruit drug users with social characteristics similar to their own. Another study [29] highlighted that the peer-recruitment approach can also be harmful to communities, as it can increase the risk of coercion when it is used without developing sustainable community partnerships, without proper support for communities, or when it poses threats to the confidentiality or privacy of participants.

Further, an important element in a subset of the CBPR studies we reviewed is their emphasis on ensuring the voices of diverse members of drug user communities are represented in research practices. Overall, six out of 25 studies (citations listed in Table 3) highlighted the importance of attending to the socio-cultural nuances, norms, and needs of diverse communities of people who use drugs. It is important to note that only six publications included descriptions of participants and provided contextual information on the participants’ background (e.g., demographic factors or other socio-cultural aspects of communities of people who use drugs). Table 5 represents some of the diverse groups of participants in these studies.

**Table 5:** Socio-demographic characteristics of participants in several CBPR studies with people who use drugs

Citation	Population
Baldwin et al. 2009 [43]	American Indian, Alaska Native communities
Brown et al. 2005 [45]	Women who inject drugs
Perez et al. 2009 [44]	
Salmon et al. 2010 [32]	
Perez et al. 2009 [44]	Prisoners who use drugs
Shannon et al. 2007 [41]	Substance-using women in survival sex work
Singer et al. 2008 [36]	Latino and Hispanic people who use drugs
Stewart et al. 2012 [42]	African-American cocaine users in rural U.S.

In terms of challenges specific to CBPR research with distinct communities, the studies reviewed highlight the importance of taking into account their norms, history, and cultural location. For example, research with Latino and Hispanic people who use drugs identified the following ethical challenges in CBPR, including loss of confidentiality, stigma related to HIV or drug use, fear of family or community rejection, as well as misconceptions and stereotypes [36]. A study with African-American cocaine users in rural Arkansas [42] described community-researcher collaborations, and suggested the importance of training “translators” – individuals who were well versed in the experiences of African-American cocaine users in rural communities, and those who understand the lived realities of people who use drugs and maintain consistently open avenues of communication between community members and researchers.

Similarly, research with Indigenous communities in the U.S. (American Indian and Alaska Native communities) illustrates the benefits of CBPR (collaborative relationships, development of programs in culturally acceptable ways, disseminating research findings from Indigenous perspectives), but also reminds researchers how important it is to be aware of and sensitive to the history of relationships before a community is approached [43]. In a similar fashion, another study [44] suggests that CBPR can be a valuable tool for determining the immediate concerns of prisoners, such as the receipt of high-quality and dignified health care inside and outside prisons (as this population bears significantly higher rates of Hepatitis C, HIV, tuberculosis, and other physical conditions). From a research ethics perspective, this work highlights that in building research agendas researchers must be sensitive to the priorities of marginalized populations and ensure the participation of communities impacted by the resulting policies.

Four publications focused specifically on the lives of women who use drugs [32, 41, 44, 45]. One study [44] identified that female prisoners tend to have lower incomes, lower educational levels, and higher rates of mental illness and substance abuse issues than women in the non-prison population. This study [44] also suggested in pursuing CBPR, it is essential to consider the critical ethical question regarding how to ensure that women engaged in CBPR truly benefit from this process.

Another study with substance-using women [41] examined the HIV-related vulnerabilities, barriers to accessing care, and the impact of current prevention and harm reduction^4^ strategies among women in survival sex work. This study highlights the concern of ensuring privacy and confidentiality for all participants, both of which require particular consideration in the context of drug use and sex work. Finally, one study [45] provided examples of the barriers and best practices associated with different steps in community-based collaborations with women who inject drugs. These scholars highlight the importance of overcoming stereotypes, treating each person with dignity, praising accomplishments, developing a “trusted” presence in the drug-user community, frank discussions among collaborators, and acknowledging different agendas as a means to overcome communication barriers among stakeholders in community-based collaboration.

## Theme 4: capacity building and CBPR

Five publications [32, 41, 43, 45, 46] out of the 25 that we reviewed focused on capacity building among people who use drugs and suggested that individuals who use drugs who participate in research-related activities (especially those who are affiliated with community-based agencies) should be offered employment, salaries, recognition, training, opportunities to participate in decision-making, and other types of support. Few articles contained descriptions of, or recommendations for, capacity building with drug users not already connected with community-based organizations/agencies.

For staff capacity building within drug user communities, one study [41] suggests that community-based agency staff should be compensated for all evaluation and research-related activities. They should be offered salaries and opportunities to publish while sharing the decision-making process, as it is inappropriate to expect community members to serve in volunteer roles when academic researchers receive salaries to conduct studies. More specifically, with regards to opportunities like employment, the literature recommends employing people who use drugs in both key roles at community-based agencies [32] and low-threshold employment positions, which work from a harm reduction perspective [45]. The project by Shannon and colleagues [41] described the capacity building process, where a team of substance-using women with lived experience of survival sex work was hired, trained, and supported to play an active role in guiding, developing, and conducting the research.

Another study [43] discussed engaging women who use drugs in paid employment and training them as peer interviewers on the CBPR project. This study suggested that the perspectives and knowledge gained by employing peer researchers/interviewers enhanced the capacity of the research team as well as the type of knowledge gained, while the research training provided the peer interviewers with skills and income that enhanced their capacity for engaging in future research, organizational development, and community action [43].

In another study [46] researchers provided training to people who use drugs as peer public health advocates (PHA), to bring a structured, peer-led intervention into sites where participants and their peers and drug-using social networks use drugs. This study identified that barriers for PHAs to complete their training included homelessness, and arrests for drug-related or other charges, such as theft, trespassing or loitering [46]. This study [46] also provided a recommendation to researchers and policy makers that they take into account the ways homelessness, as well as arrests for drug-related or other charges, are significant barriers for marginalized people who use drug to complete training and gain employment.

## Theme 5: pragmatics of participation and inclusion in CBPR

Nine of the 25 publications discuss the pragmatics of the inclusion of people who use drugs. Specifically, two publications [6, 39] describe the benefits of including people who use drugs in CBPR. According to one study [39] the involvement of people who use drugs in community-based research may result in the inclusion of culturally appropriate questions, a sampling design that is reflective of the community needs, and findings that take community contexts and lived experiences into consideration. Further, the literature suggests that the benefits of including people who use drugs (in particular people who inject drugs) in CBPR research can be empowerment, increased tolerance of drug using behaviours, reduction of stigma, and increased social support networks [40].

When the topic of exclusion is discussed, three publications highlight (and at times critique) how and why researchers exclude participants who use drugs who are deemed too intoxicated to participate in research (Table 6). One study [26] suggests that excluding injection drug users from clinical trials is unacceptable unless there is strong evidence to support exclusion (e.g., threat to safety). Another study [47] critiques how researchers sometimes exclude intoxicated participants when they are deemed “too intoxicated” to participate. Finally, a third study [30] highlights how many researchers often exclude people from their studies who test positive for illicit substance use, based on reasons such as possible non-compliance with research protocols (e.g., follow-up appointments), even though research suggests that the use of CBPR to enroll people who use drugs can achieve a 95 % retention rate [30].

**Table 6:** Examples of justifications for exclusion of people who use drugs from research

Citation	Justification for exclusion
Aldridge and Charles 2008 [47]	Describes assumptions held by researchers that exclusion is justified if a participant is deemed “too intoxicated” to participate (based on biochemical or bio-behavioural screening).
DuBois et al. 2011 [48]	Describes how financial resources may limit the number of community members who can be included or involved in research.
Levine et al. 1991 [26]	Suggests there is strong evidence to support exclusion if a drug-using participant is ‘a threat to safety’.
Striley et al. 2008 [30]	Describes assumptions held by researchers that exclusion is justified because participant may become non-compliant with research protocols or follow-up appointments

Only two publications [32, 48] gave specific recommendations concerning how to increase the inclusion of people who use drugs in CBPR. One study [32] critiqued the class-based and ableist biases implicit in some CBPR projects, such as, that all partners must demonstrate equitable participation by remaining actively involved in all phases of the research process. Instead, researchers recommended accommodating people in research who want to participate at different times and in ways that shift in accordance with their health status, drug use patterns (and consequences), housing concerns, and other aspects of their lives. Similarly, one study [48] suggested that while many members from a community may wish to engage with researchers, available resources limit the number of community members who can be engaged. As such the recommendation is that CBPR researchers should always remain critical and reflexive of how many, and which individuals from the community, are provided with a voice and power to make key decisions in research.

## Discussions

### Summary of findings

Participant compensation was the ethical issue most commonly addressed in the literature included in this scoping review, and a consensus emerged that people who use drugs should not receive lesser compensation (or restrictive compensation, such as gift cards) solely because they are known to use drugs non-medically. However, these articles identified the need for clear communication with communities and potential participants about compensation, given that active drug users often live in extreme poverty and thus compensation practices perceived to be unfair can be particularly problematic. Under the theme of perceptions of people who use drugs related to research, studies suggested that people who use drugs often participate in research for altruistic reasons or to help their own communities, in addition to potential personal benefits. Perceived negative aspects of participation often related to fear of research related risks, and concerns about the whether the research would have real-world positive impacts. Taken together, these findings point to the importance of ensuring that research projects balance risks and benefits to communities of people who use drugs, as well as individual people who use drugs, and that community-level as well as individual risks and benefits are clearly considered and communicated.

Peer recruitment and representation was another thematic area that emerged, the latter primarily in relation to Indigenous peoples and communities of color in the United States, but also women, sex workers, and prisoners. Peer recruitment was viewed as integral to recruiting large and diverse samples of people who use drugs, but also presented ethical challenges regarding support for recruiters, coercive recruitment, and participant confidentiality.

Under the theme of capacity building, low-threshold and flexible employment and training for people who use drugs was identified as an important component of ethical CBPR practice. Finally, studies related to pragmatics of participation and inclusion in CBPR suggested that excluding people from research on the basis of drug use at the time of recruitment is usually unethical, but that inclusion needs to be balanced with the ability to give informed consent while under the influence of drugs.

Reflections on successful CBPR projects with people who use drugs led to recommendations for flexible and accommodating practices that are responsive to the oft-chaotic living conditions of low-income people who use drugs, so that individuals are not entirely excluded from study involvement if they are deemed (or deem themselves) unable to participate at a particular time. Such practices may help to resolve the ethical and pragmatic dilemmas that arise from policies regarding study participation for people who are actively or currently using drugs.

### Research gaps

The 25 articles included in this scoping review addressed issues relevant to the nexus of ethics, CBPR, and people who use drugs in five thematic areas. However, we found no studies explicitly focused on ethical issues in the context of community-based participatory research with people who use drugs^5^. Moreover, few studies addressed ethical issues specific to *community-based* participatory research with people who use drugs. Given the recent proliferation of resources specific to peer involvement in CBPR, this is a noteworthy gap. Themes that were most specific to CBPR with people who use drugs (e.g., capacity building) often focused on their involvement as peer recruiters or study staff. Yet, most community members will be involved in a CBPR study primarily as participants. Nevertheless, a CBPR approach raises additional ethical dimensions across all levels of involvement (e.g., around personal disclosure to peer researchers, intra-community conflicts) [11, 17, 19]. While these topics are becoming more visible in the body of work related to community-based research ethics, they are unaddressed in the reviewed literature specific to research with people who use drugs. Most studies were conducted in or focused on Canada, Australia, the United Kingdom, and the United States, and thus additional knowledge is needed regarding ethical issues for CBPR with people who use drugs in settings where drug use is more or less criminalized or stigmatized (e.g., in the few countries or regions that have decriminalized some or all personal drug use, as well as in settings where drug-related offenses are punishable by forced “treatment”, corporal punishment, or death).

### Limitations of our approach

While our search terms were developed in consultation with a research librarian and selected to include the broad scope of terms used in the literature, there may have been relevant peer-reviewed articles that were not captured by these terms. In addition, our search of the grey literature was not exhaustive or systematic.

### Conclusions

Many ethical issues pertaining to CBPR are common across marginalized populations. For instance, CBPR with people who use drugs may be similar to CBPR endeavors with other marginalized groups with respect to ethical imperatives and challenges to community engagement and sustained involvement. However, this scoping review revealed some unique ethical considerations for CBPR with people who use drugs that have been under-addressed to date. With respect to the pragmatics of inclusion and exclusion in research, Aldridge & Charles [47] address the ethical imperative to ensure a balance between protecting the autonomy of people who use drugs (who may be more likely to consent when intoxicated) and justice in relation to extending the benefits of research participation. However, their recommendations explicitly assume that intoxication is “relatively transitory”, and thus, it is unclear how they might apply to people who use drugs on a daily basis. In addition, no specific guidelines exist for the bio-behavioural assessment and screening of intoxicated participants, partially because both biochemical and behavioural methods for identifying intoxication are problematic. Instead, researchers should seek to devise research protocols that acknowledge intoxication and protect research participants by offsetting risks and potential harms to participants [47]). More empirical research is needed to understand the complexities of consent for people who use drugs frequently or daily. In addition, policies and practices that exclude the most intoxicated individuals may, consciously or not, result in research findings that do not reflect the realities of those most at-risk of poor health and social outcomes, and may limit their access to material and other benefits of CBPR (compensation, training, peer support).

Articles included in this scoping review did not directly address competing or unclear definitions of “community” that might complicate the ethical engagement of people who use drugs in CBPR. For example, there are many clearly documented benefits of involving both active users and former users in various types of health promotion efforts, as well as the substantial role of former users in drug user treatment and research [42, 46]. Elsewhere, Roy [12] distinguishes between CBPR with people who use drugs versus CBPR about drug use with non-using members of minority communities; these divergent approaches to research were not distinguished, or possibly conflated, in some articles (e.g., [43]). At issue in this distinction is whether drug use is treated as a behaviour, or as a characteristic that, at least in some cases, defines an identifiable community that must be engaged in community-based research about drug use. These questions reflect broader discussions and tensions concerning definitions of *community* in the context of CBPR [49, 50], which have specific implications in the context of drug use. For example, under the theme of capacity building, most articles assumed that people who use drugs would be engaged in research through community health organizations that they are affiliated with (e.g., HIV/AIDS service organizations, community health centres), or that the organizations themselves would be the “community” research partners. Yet, such organizations are typically staffed and directed by people who do not identify as active drug users. Where they exist, it seems that drug user-led networks and organizations would be well-suited to represent community interests in research. However, such groups may require additional support, time, training, and material resources in order to participate in research; they may also have more explicitly political aims and activities that conflict with some researcher agendas. We believe that more attention should be paid to defining and mobilizing communities of people who use drugs, with an ethical orientation towards supporting drug user self-organizing (recognizing that participation in research may help fledgling organizations to develop capacity) [7, 51, 52].

## Availability of data and materials

The data supporting the conclusions of this article is available in the LabArchives repository.

doi:10.6070/H4S75DBB. https://mynotebook.labarchives.com/share/cbrethicspwud/MjIuMXwxMzgxMDIvMTcvVHJlZU5vZGUvODMyNTY0NTkxfDU2LjE=

## Abbreviations

CBPR: community-based participatory research
PWUD: people who use drugs
REB: research ethics board

## Footnotes

1 Community-based participatory research (CBPR) is a term used throughout this paper. Other variations of this term (with their own distinctions) include: community-based research (CBR), Community-Engaged Research (CEnR), and action research (see Wallerstein and Duran [8] for further background).

2 Other variations of the term Research Ethics Board (REB) are Research Ethics Committee (REC), and Institutional Review Board (IRB) – the latter term primarily used in the USA.

3 This project did not require research ethics review under the guiding document for research in Canada – known as Tri-Council Policy Statement (TCSP2). TCPS2 article 2.5 provides an exemption for activities that do not meet the threshold for the Canadian definition of research but may use techniques similar to those employed in research. For example, quality assurance, program evaluation activities and other activities used exclusively for assessment, management or improvement purposes – such as the activity we did. No personal or sensitive data was collected through these consultations. University of Toronto Research Ethics Board was consulted and they deemed this projected not to require ethics review. Given that this project did not meet the definition of research and was minimal risk, the consultants form the communities were informed verbally about the nature of their involvment. The team decided not to collect any personal nor sensitive information given that collecting that information may put community consultants in harm's way and expose them to risk.

4 Harm reduction is a non-punitive, pragmatic approach to drug use, which acknowledges that people engage in behaviors that may harm their health, and seeks to reduce these harms [45].

5 One study was published since our search: Strickland JC, Stoops WW. Perceptions of research risk and undue influence: Implications for ethics of research conducted with cocaine users. Drug Alcohol Depend. 2015. doi: 10.1016/j.drugalcdep.2015.09.029.
